# Towards halal pharmaceutical: Exploring alternatives to animal-based ingredients

**DOI:** 10.1016/j.heliyon.2023.e23624

**Published:** 2023-12-13

**Authors:** Yedi Herdiana, Ferry Ferdiansyah Sofian, Shaharum Shamsuddin, Taofik Rusdiana

**Affiliations:** a Department of Pharmaceutics and Pharmaceutical Technology, Faculty of Pharmacy, Universitas Padjadjaran, Sumedang, 45363, Indonesia; b Department of Pharmaceutical Biology, Faculty of Pharmacy, Padjadjaran University, Sumedang, 45363, Indonesia; c Halal Food Pharmaceutical and Healthcare Society, Faculty of Pharmacy, Padjadjaran University, Sumedang, 45363, Indonesia; d School of Health Sciences, Universiti Sains Malaysia, 16150, Kubang Kerian, Kelantan, Malaysia; e Nanobiotech Research Initiative, Institute for Research in Molecular Medicine (INFORMM), USM, 11800, Penang, Malaysia; f USM-RIKEN Interdisciplinary Collaboration on Advanced Sciences (URICAS), 11800, USM, Penang, Malaysia

**Keywords:** Animal by-products, Active ingredients, Excipients, Halal, Porcine

## Abstract

Halal is a crucial concept for Muslim consumers regarding consumed products, including pharmaceutical ingredients, which are essential in modern medicine. To address the issue of using porcine-sourced ingredients in pharmaceuticals, it is essential to search for halal alternatives derived from poultry, animal by-products from meat processing, marine sources, and plants. However, the complexity of this problem is further compounded by the rapid advances in innovation and technology, which can lead to adulteration of ingredients derived from pigs. Other challenges include the sustainability of alternative materials, management of waste or by-products practice, halal awareness, certification, government policies, religious adherence of consumers, food suppliers, marketers, and purchasing of products. The importance of halal and non-halal problems, specifically in the context of pharmaceutical materials, is still rarely discussed, including alternatives derived from poultry, animal by-products, marine sources, and plants. Due to the increasing global population, there is a growing need to increase awareness and concern among Muslim consumers for halal products, including pharmaceuticals. Therefore, this research aimed to investigate the importance of halal and non-halal issues in pharmaceutical ingredients, the potential impact on the Muslim community, as well as opportunities and challenges in the search for alternative ingredients.

## 1 Introduction

The concept of halal is beyond national and Islamic boundaries, evolving into a global concern for food, cosmetics, drug manufacturers, and consumers. Furthermore, its significance has transcended local and religious implications, prompting widespread attention in the production and consumption of various products [1,2]. Halal pharmaceutical and cosmetic products are gaining increased awareness and demand among the 2.4 billion Muslim consumers globally [3,4]. Based on estimation, the global halal market is expected to expand at a compound annual growth rate of 6.8 % until 2024 [5]. This concept emphasizes the importance of adhering to approved Shariah procedures in products that are consumed [6,7] and the need for ‘toyyib,’ which requires meeting high-quality standards [4,5,8]. The implementation of a halal assurance system throughout the production process is crucial to ensure the delivery of products that meet quality standards [9]. In the Islamic context, this concept revolves around the permissibility of consuming specific products, as non-halal items can adversely affect both the health and behavior of consumers [10,11]. The issue of halal has gained global attention, particularly among conscientious Muslims who are careful of consuming a wide range of products, including food, beverages, insurance, leather goods, pharmaceutical, and cosmetics. Islamic teachings strongly advocate for the consumption of halal products [12]. In Islam, the promotion of permissible and lawful remedies for treating illnesses is evident through authentic Hadiths. The Prophet Muhammad (peace be upon him) once stated, “There is no disease created by Allah that He also has created its treatment.” This Hadith from Sahih Bukhari underscores the belief that Allah has provided remedies for every illness, thereby showing the importance of seeking treatment. Consequently, Muslim communities are encouraged to explore healthcare options in line with halal principles, ensuring that the medicines used are free from any prohibited or doubtful substances. The incorporation of these Hadiths in the background section provides a formal and religious perspective on the significance of halal medicines within Muslim communities. By adhering to healthcare practices with Islamic teachings, individuals can uphold halal principles and contribute to community well-being [13].

The potential of tapping into a large population as a market share can only be fully realized through precise engineering and design. The implementation of mandatory halal certification for imported products, particularly in the food, pharmaceutical, and cosmetics sectors, can effectively address import-related issues while simultaneously stimulating demand. This strategic method offers significant advantages to countries with substantial populations, enhancing their position in the global market [3,14,15]. However, the proliferation of standard-setting and certification bodies can create a complex operating environment for halal pharmaceutical companies [16,17]. Despite these challenges, certification remains important to cater to the needs of the extensive community and potential market by fostering the domestic development of halal products. The decision of the Indonesian government to postpone proposed changes has shown the significance of collaboration and communication among stakeholders to ensure the effective implementation of halal standards in the pharmaceutical industry [7,[18], [19], [20]].

The approval of halal labeling is the first step in meeting the demand for halal products [21]. To enhance consumer awareness and increase the demand for halal products, there is a need to provide transparent labeling on products. The inclusion of halal logo, including those for over-the-counter medicines, can also empower consumers to make informed selections about their purchases [21,22]. The development of products prioritizing Muslim consumers and a “halal-first” method will be essential for future growth. This is because access to medicines in accordance with one's beliefs is a fundamental right. However, the proposal for mandatory halal labeling on pharmaceutical products in Indonesia has encountered resistance from the International Pharmaceutical Manufacturing Group (IPMG), citing a lack of incentives for investing in halal product manufacturing due to the limited market size of the country. This necessitates the search for a solution that meets the needs of both the industry and consumers through collaboration and communication among stakeholders.

Pharmaceutical raw materials originate from diverse sources, including animals, plants, and synthetic materials [23]. Although certain drugs, such as heparin and insulin, are derived from porcine-based sources [24,25], several pharmaceutical raw materials are obtained from non-animal origins. For some consumers with religious or cultural constraints, the use of animal-derived pharmaceutical materials poses a significant concern [[26], [27], [28], [29]]. Consequently, the development of alternative materials as substitutes is essential to meet the growing demand for halal and vegetarian-friendly pharmaceutical products.

Excipients are inert components used in pharmaceutical formulations for various purposes, including binding, lubrication, and stabilizing active pharmaceutical ingredients [27,[29], [30], [31], [32]]. Although some excipients might be obtained from animal sources, such as pork [[33], [34], [35]], alternatives to pork-based materials, including those sourced from plants or created synthetically, are still in existence [23,31,36,37]. Pharmaceutical companies can select these alternative excipients to accommodate consumers with religious or cultural restrictions regarding the use of pork-derived materials.

In the development of halal pharmaceutical, substitute materials must be explored, adhering to halal criteria and minimizing the use of critical ingredients [12,[38], [39], [40], [41]]. Regulatory bodies might also mandate halal certification to ensure that products and excipients used adhere to the requirements of specific markets or consumers. Consequently, pharmaceutical companies are encouraged to disclose the origins of their materials and work on providing alternatives for individuals who cannot consume pork-derived products due to their beliefs or preferences.

This review underscores the challenges and prospects associated with discovering halal alternatives for pharmaceutical components, particularly excipients. It emphasizes the importance of collaboration among regulatory authorities, showing the need for educating healthcare professionals about the development of halal pharmaceutical. Therefore, this research aimed to investigate the issue of pork-based ingredients and fulfill the demands of Muslim consumers by increasing awareness and advocating for halal alternatives sourced from various origins such as poultry, halal animal by-products, marine sources, and biotechnology. The results are expected to provide advancement in the search for halal alternatives within the pharmaceutical industry to cater to the diverse needs of consumers.

## 2 Drug-animal derived

### 2.1 Halal

According to the Qur'an, pork is deemed impure and unsuitable for human consumption, as stated in the following verse: “Forbidden to you (for food) are dead meat, blood, the flesh of swine … " (Qur'an [Al-Maidah verse 3]). The prohibition of pork is reiterated in several cases throughout the Qur'an, including Qur'an [Al Baqarah verse 173], Qur'an [Al An'am verse 3], Qur'an [Al A'raf verse 145], and Qur'an [Ibrahim verse 115]. However, the Qur'an acknowledges the principle of necessity, which permits the consumption of prohibited substances in situations that may lead to life-threatening conditions or organ damage, specifically in the medical field. In this research, the legitimacy of various porcine-derived products was explored based on the prospective circumstances of each case [42].

The primary sources for halal principles are the Quran and the Sunnah of the Prophet. The four major Sunni schools of Islamic jurisprudence have varying methods to determining halal sources [43]. The Hanafi school has a broad perspective, which allows a wide range of animal products unless explicitly prohibited. The Maliki school follows local customs and considers only items explicitly mentioned as halal in the Quran and Sunnah. Meanwhile, the Shafi'i school relies on evidence from the Quran, Sunnah, and analogical reasoning. The Hanbali school prioritizes textual evidence and adopts a stricter interpretation. These differences reflect variations in legal opinions within Islamic jurisprudence but ensure adherence to Islamic principles and promote the concept of halal [44].

Production of halal products adheres to several key principles:

1) All ingredients must be safe for consumption and free from ethanol, blood, pork, and parts of carnivorous and omnivorous animals, including human parts. In Indonesia, The MUI Fatwa permits the use of non-khamr sources of ethanol, such as synthetic or industrially fermented ethanol, with specific restrictions. Furthermore, it allows a tolerance for ethanol content in beverages below 0.5 % when it is medically harmless. This guidance ensures that halal-certified products can include permissible ethanol while maintaining consumer safety and adherence to Islamic principles.
2) Stringent hygiene measures must be applied to minimize any contamination with potentially toxic, ritually unclean, or impure ingredients.
3) The entire production process, from cultivation to distribution, must adhere to Sharia law and comply with halal standards.
4) Halal production must be physically separated from non-halal production to prevent any mixing of the two.
5) Any potential cross-contamination between halal and non-halal ingredients and products must be prevented [45].

Islamic dietary laws mandate that halal animals used for food or medicine must pass through a specific method of slaughter, known as halal slaughter. This method entails a swift cut to the throat with a sharp knife, severing the jugular veins and carotid arteries while leaving the spinal cord intact. Specifically, a halal animal must be alive and in good health at the time of slaughter, and the name of Allah must be invoked before making the cut. By adhering to these guidelines, the animal is considered halal and permissible for consumption or use in medication by Muslims. Although the halal certification process for pharmaceutical products shares some similarities with the standard process, there are significant distinctions [6,38,46,47].

Each drug must be assessed independently due to the technically complex and distinct nature of pharmaceutical products, considering its formulation, methodology, and intended use case [7]. Halal pharmaceutical ingredients are constituents derived from plants, soil, water, and animals slaughtered according to Islamic law, marine animals are considered halal, and synthetic materials are safe for consumers and unadulterated with filth (najis). As a rule of thumb, manufacturers must secure halal certification for each ingredient from suppliers [5].

Istihalah is an Islamic concept that entails the transformation of a substance by mixing it with others to obtain new products with altered form and content [48]. When a prohibited substance passes through transformation, the governing rule can change according to Shariah. For example, when alcohol naturally or through chemical processes transforms into vinegar, istihalah becomes relevant. Several important considerations are as follows: 1) The process does not render impure things pure unless transformed and is not inherently impure. However, vinegar made from wine is considered pure. 2) Processes using impure growth media require purification, while 3) ingredients derived from swine or its derivatives are generally prohibited. Scholars may have varying interpretations, making it necessary to seek guidance [[48], [49], [50]].

### 2.2 Pharmaceutical dosage form

Pharmaceutical products are substances designed for diagnosing, treating, mitigating, curing, or preventing disease [29,51]. These products are produced using various materials derived from plants, animals, chemicals, and minerals through manufacturing processes ranging from simple to highly complex [[52], [53], [54]]. Furthermore, pharmaceutical products are categorized based on their pharmacological effects and intended therapeutic uses [55,56]. Before approval for production, rigorous evaluation must be carried out by government regulatory agencies to ensure the safety and effectiveness of these products. The manufacturing process is subject to stringent quality control measures, including a range of “acceptable operating conditions” to maintain consistency and quality while accommodating variations in materials and production processes [9,57,58].

Raw materials serve as the basic components for manufacturing medicines and supplements. In the pharmaceutical industry, raw materials are categorized into two, namely active ingredients and excipients.

#### 2.2.1 Active ingredients

Active ingredients are components intended to provide pharmacological activity or another direct effect in diagnosing, curing, mitigating, treating, preventing disease, and influencing the structure or function of the human body and other animals. These ingredients can be derived from animals and plants [23], including pigs. Moreover, when active ingredients are sourced from pigs or non-halal animals, the resulting products are considered haram (forbidden). When active ingredients are obtained from halal animals, slaughtering must be carried out in accordance with Islamic principles [12,44,59].

#### 2.2.2 Excipients

**Fig. 1:**
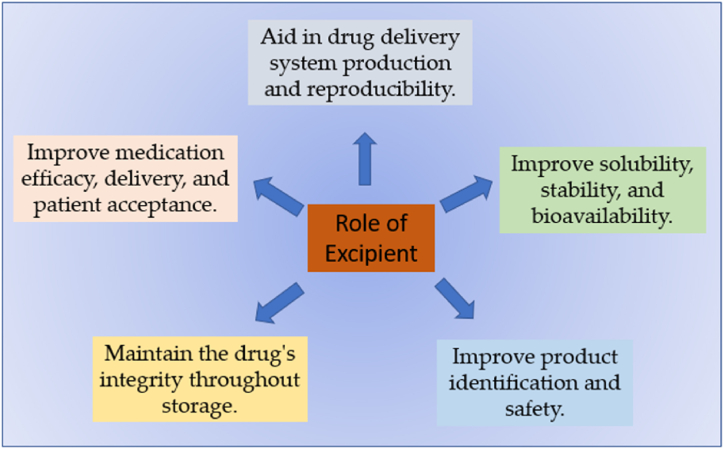
Role of excipients in drug formulation.

Excipients are inactive substances such as coloring agents, preservatives, and fillers that serve as carriers for a drug or other active components [31,51]. Furthermore, excipients must be halal, but their role may not be as prominent as raw materials. In synthetic drugs and supplements, specific attention must be given to critical excipients components. For example, tablet coatings containing gelatin require verification to ensure that the content is sourced from halal animals and processed in accordance with Islamic guidelines. The role of excipients in drug formulation is shown in Fig. 1. The requirements for excipient pharmaceutical products are as follows:

1. Biologically stability should be standardized and approved by regulatory bodies derivative.
2. Easily available, cost-effective, high flexibility, thermo stable, sterilizable
3. Chemically, physically, therapeutically inert, nontoxic, compatible
4. Odorless, tasteless, elegant, pharmaceutically acceptable

**Fig. 2:**
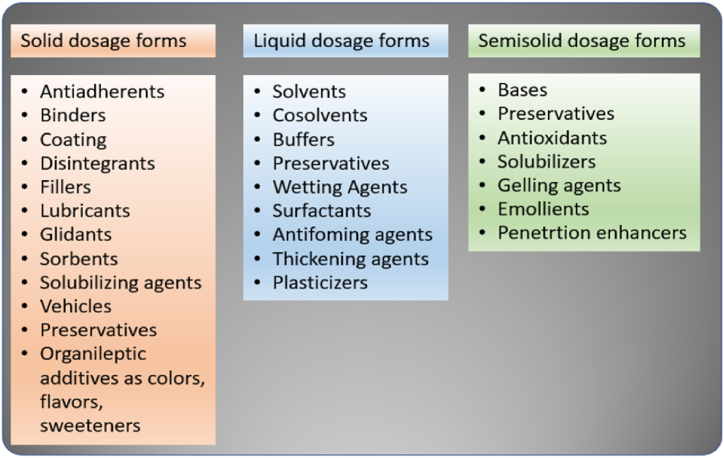
Excipients function in various pharmaceutical preparations.

Islamic law prohibits the consumption of animal-derived ingredients that are not slaughtered according to Islamic guidelines. This prohibition directly conflicts with various pharmaceutical products, including solid, liquid, and semisolid dosage forms, as presented in Fig. 2. One common ingredient used in capsule shells for drug delivery is gelatin, a protein obtained from the skin, ligaments, tendons, as well as bones of pigs and cows [10,60,61]. Several medications, including amoxicillin, omeprazole, warfarin, prednisolone, oxinorm, and heparin, contain animal-derived (porcine or bovine) components, with gelatin serving as a widely used vaccine stabilizer [28]. Another example is magnesium stearate, a common ingredient in many tablets, which can be obtained from plants or animals [62].

Synthetic drugs and supplements, primarily produced through chemical processes, have a minimal risk of containing non-halal contaminants. However, in the case of herbal medicines, special considerations must be given to the extraction process to ensure halal status. This is particularly critical when the extraction includes animal materials, which require verification.

### 2.3 Porcine-derived pharmaceutical raw material

According to Islamic law, Muslims are required to consume products that are both halal and healthful, considering ingredients and the entire drug manufacturing process [28,29,63]. Pork is generally prohibited, but exceptions can be made in cases of necessity when there are no viable non-porcine alternatives with comparable quality and efficacy [8,14,[64], [65], [66]]. The level of necessity, ranging from elective to absolute, is also a crucial aspect. This is because, in situations where non-porcine alternatives are unavailable, the urgency of a medical show may justify the use of a prohibited porcine biomaterial [15,67].

Animal models, particularly pigs, are a valuable tool in biomedical research. Pigs share several anatomical, genetic, and physiological similarities with humans, leading to their relevance for various investigations, including obesity, female health, cardiovascular disease, nutritional research, and communicable disease [68,69]. Specifically, their close approximation to the human body is advantageous in research areas such as tissue engineering, imaging, surgery, chemotherapy, and radiation research, where smaller animal models, such as mice, may yield less accurate results. Furthermore, the use of numerous established cell lines derived from various swine tissues has significantly facilitated various in vitro research. Pigs also serve as robust models in cancer research, enabling preclinical imaging research, preclinical drug screening, and the exploration of several interventional therapies such as hyperthermia, radiation, and photodynamic therapy. As resources related to porcine genomics and epigenetics continue to advance, their application in biomedical research requires further expansion [70]. Pigs also possess a multitude of anatomical and physiological similarities to humans, confirming their status as highly valuable animal models for translational research. Based on the ongoing advancements in genetic engineering methods, the potential to harness pigs as precise and predictive models for human diseases is rapidly evolving. The development of Porcine Precise and Predictive (PPP) models, replicating human disease mechanisms and molecular targets at a molecular level, offers a promising platform for the in-depth research of disease pathogenesis and the assessment of therapeutic interventions [71].

**Fig. 3:**
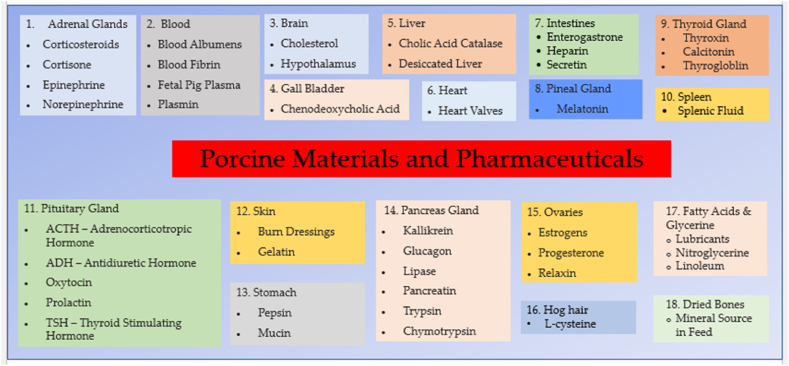
Porcine-derived material and Pharmaceutical.

Extensive research is currently ongoing to determine the most reliable tissue sources and the optimal physical, enzymatic, or chemical processes for obtaining the best products. Several pharmaceutical products derived from porcine sources are currently in use, as shown in Fig. 3. Some examples include:

1) Insulin, porcine insulin is structurally similar to human insulin and is used as a replacement therapy for patients with diabetes [72].
2) Collagen is a protein used in various medical and cosmetic products, including wound dressings, tissue engineering, and injectable fillers. Furthermore, porcine collagen is similar in structure to human collagen and is often used as a substitute [73,74].
3) Gelatin is a protein derived from collagen found in pig skin, bones, and tendons. This protein is commonly used as a gelling agent, thickener, and emulsifier in solid and liquid oral dosage forms as well as in food products [12,27,59]. Gelatin is also frequently used in capsules, but alternatives derived from plants and algae are available for vegetarian and vegan consumers [12,27,59]. Moreover, halal certification is essential for gelatin sourced from non-halal animals, while those obtained from bovine and fish sources are considered permissible. Capsules are customizable and enhance patient compliance due to their compact size and ability to mask undesirable tastes.
4) Lactose is a sugar derived from whey and a by-products of cheese production, which is commonly used as a filler, binder, and diluent in tablet and capsule formulations.
5) Stearic acid is a fatty acid derived from pig fat, commonly used as a lubricant and release agent in tablet formulations [75].
6) Lard is a fatty substance derived from pig fat, which is commonly used as a cooking oil and a component of margarine and other spreads.
7) Heparin is a medication used for blood thinning, extracted from the intestinal mucosa of pigs, which shares a structural resemblance to human heparin. Presently, a staggering 100 metric tons equivalent to 1.5 billion doses of heparin, are used globally each year, necessitating tissue from approximately 700 million pigs. Although there might be alternative treatments available in certain cases that do not rely on pig sources, suitability or effectiveness is not guaranteed. In some situations, there may be no viable substitute for heparin derived from pigs.
8) Enzymes are porcine-derived, such as trypsin and pancreatin, which are used in various digestive and metabolic disorders.

The use of porcine-derived materials in pharmaceutical products must be evaluated regarding safety, efficacy, and ethical considerations. Furthermore, the ethical implications of using animal-derived materials are required.

### 2.4 Challenge for new candidate pharmaceutical substitution

The challenge for a new candidate to substitute pharmaceutical excipients is to ensure that the replacement does not adversely impact the safety, effectiveness, or stability of the drug products [[76], [77], [78], [79]]. Excipients are non-active ingredients used in drug products to improve their physical and chemical properties, enhance stability, and facilitate administration [30,80,81]. Substituting excipients can offer advantages such as cost reduction, improved flexibility in the supply chain, and addressing regulatory concerns [31,82]. However, substitution is expected to pass a thorough evaluation to guarantee the safety or effectiveness of the drug products. An intriguing case research is the use of gelatin in essential pharmaceutical applications to shield drugs from detrimental factors such as light and oxygen. Gelatin can be applied in the formulation of both soft and hard capsules [60,61]. The development of substitute development poses challenges in the areas stated below:

1) Cost-efficiency

In comparison to alternative excipients, gelatin is the most cost-efficient option. The raw materials for first-generation Hydroxypropyl Methyl Cellulose (HPMC) hard capsules incur approximately four times the cost of gelatin, and the manufacturing of HPMC capsules is significantly more expensive.

2) Machinability

Gelatin exhibits a high level of machinability, showing its ability to withstand mechanical processes without requiring additional ingredients or machinery. In contrast, HPMC shows lower machinability, as first-generation HPMC requires secondary gelling agents, while second-generation only demands machine adaptation.

3) Mechanical resistance

Capsules must possess enough strength to endure mechanical stress, as any weaknesses can result in defects. In terms of mechanical resistance, gelatin surpasses HPMC by a significant margin. Regarding soft capsules, gelatin, and modified starch have shown comparable strength.

4) Oxygen permeability

Some active ingredients are sensitive to oxidation due to their complex profiles. This phenomenon makes capsules the preferred option for these formulations, providing superior protection. Gelatin has become the optimal excipients when considering oxygen-sensitive active ingredients. However, HPMC shows a higher potential for oxygen penetration, requiring the use of additional ingredients such as antioxidants, which increases total costs. Consequently, manufacturers tend to opt for gelatin capsules when oxygen sensitivity is a factor.

5) Water permeability

Another critical consideration is moisture penetration, which has varying effects depending on the capsule type. Although HPMC possessed a lower water content compared to gelatin, the superiority in terms of water permeability remains inconclusive.

6) Scalability

Scalability is significantly important in the pharmaceutical industry, particularly concerning the development and manufacture of excipients. The ability to produce excipients on a large scale without compromising quality or consistency requires rigorous selection of raw materials, process optimization, and stringent quality control measures. Additionally, as the demand for a particular drug rises, the quantity of excipients required also increases. This shows the importance of designing a production process that can seamlessly accommodate volume changes without significant disruption. Regulatory compliance is also a critical factor, as the production of pharmaceutical excipients must adhere to stringent quality standards and regulations to safeguard patient safety [83,84].

## 3 Efforts made to replace porcine-derived

The use of animal-derived materials in medicine poses challenges for patients with religious or cultural beliefs against certain animal products or food allergies [19,35]. Although there are some available alternatives, regulations and guidelines regarding the disclosure of animal-derived components in medications differ from one country to another [[33], [34], [35]]. Consequently, continuous research and development of substitutes are essential to ensure the availability of safe and efficacious medicines for patients. Several initiatives are currently in progress to enhance patient access to information, which requires healthcare professionals to remain well-informed for the delivery of optimal care.

### 3.1 Halal‐Based Ingredients for substitution

**Fig. 4:**
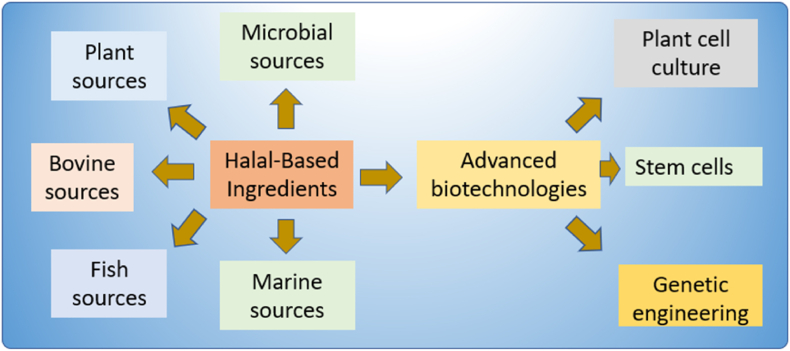
Halal‐Based Ingredients for substitution.

Alternative sources for pharmaceutical ingredients that adhere to halal guidelines are readily available. These include chitosan derived from microbial or plant origins, insulin produced through recombinant deoxyribonucleic acid (DNA) technology, collagen obtained from bovine, fish, or plant origins, and heparin derived through bovine or fish sources [12,23,37], as illustrated in Fig. 4. However, the use of non-halal growth media for microbial cultivation raises concerns regarding the permissibility of the final products for Muslims. This necessitates seeking guidance from knowledgeable sources to determine the consistency of these products with halal requirements. Muslim communities should also remain vigilant about the origin and composition of the media used in pharmaceutical production to make informed selections in accordance with their religious beliefs and dietary restrictions [85]. These alternative sources offer advantages such as the production of substantial quantities of biomolecules, high yields, and cost-effectiveness while promoting sustainable and ethical practices within the pharmaceutical industry.

Biotechnology offers promising solutions to the challenges associated with developing halal-compliant plant-based excipients for pharmaceutical and dietary supplements [43,52,59]. Advanced biotechnology, such as plant cell culture and genetic engineering, are being explored for more sustainable and efficient production of excipients [36,37,86]. Technological advancements have further expanded the range of alternatives, including the use of stem cells for insulin production [87,88]. However, the development of these excipients still presents several challenges, including sourcing halal-compliant raw materials and adhering to regulatory requirements. These excipients, which are non-active ingredients used in drug and dietary supplement formulations, represent an area of interest and innovation within the industry [39,51]. Several investigations have explored various halal-compliant plant-based excipients, such as starches, cellulose, gum Arabic, pectin, and alginates, potentially enhancing the stability and delivery of the final products [5,26,61].

**Fig. 5:**
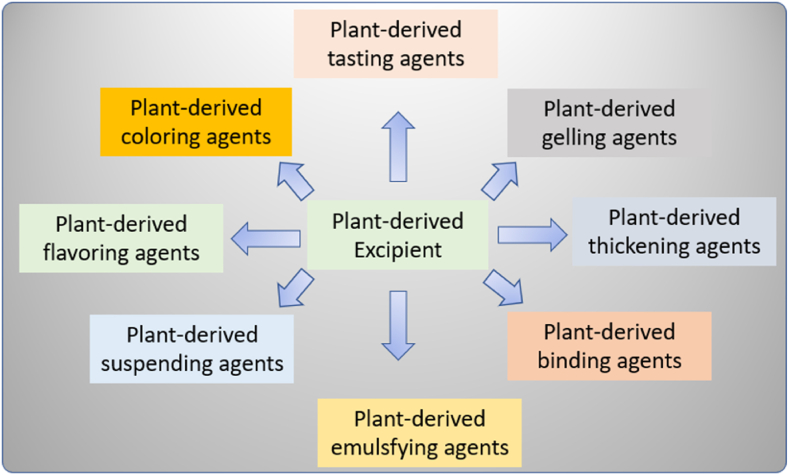
Plant-derived excipients.

The use of halal-compliant plant-based materials in pharmaceuticals, nutraceuticals, and dietary supplements is increasing due to their natural properties, biocompatibility, and biodegradability [11,17,26,29]. Plant-based materials also offer the advantage of producing 100 % halal ingredients and are being explored as alternative sources for anti-inflammatory therapy, as presented in Fig. 5. However, the safety, efficacy, and ethical implications of plant-based materials must be thoroughly evaluated. Essential oils derived from plants have gained attention for their potential pharmacological and therapeutic properties, containing active compounds with diverse health benefits. Recent research into the development of drugs from essential oils and other plant-derived compounds is expected to yield successful results in natural compounds for medical treatments [20].

Collagen extracted from fish sources has gained significant attention due to its nutritional and functional advantages across various industries [12,26,63,89]. However, production has limitations, as the most promising processes for achieving high-quality collagen can be quite costly and time-consuming. For certain Muslim communities, the use of fish as a source of collagen may not be considered halal-compliant. Consequently, efforts are being carried out to explore halal-compliant sources of marine collagen, such as those derived from shellfish or other non-prohibited marine resources [5,63,89]. The use of marine collagen in the pharmaceutical and food sectors has shown promising outcomes despite the cost and production challenges. For instance, fish collagen finds widespread application in medical treatments, including its use in collagen dressings for wound healing in dentistry and surgery. Another frequently used ingredient, namely gelatin, is predominantly sourced from non-halal sources, posing a challenge for the halal industry. Although marine gelatine is considered halal, its production remains relatively limited [90,91].

### 3.2 Transition from biological sources to synthetic agents

Drug development has experienced significant advancements in recent years, with medications being derived from animal and plant compounds. Several novel methods of manufacturing and developing drugs have been discovered due to the progress in synthetic chemistry and recombinant technology. Halal-compliant synthetic excipients have been formulated as alternatives to porcine-derived materials and other products, which can be produced through chemical synthesis or biosynthesis [54,92]. Chemically synthesized excipients include polyvinylpyrrolidone (PVP), polyvinyl alcohol (PVA), and polyethylene glycol (PEG) [51], while biosynthetically-derived are microbial-derived polysaccharides and polymers. However, a thorough evaluation of safety, efficacy, and ethical considerations is essential, along with careful adherence to regulatory compliance and halal certification requirements [43,93].

The halal status of synthetic excipients depends on the source of the initial materials used during synthesis and the final purification process. To determine this status, halal certification organizations have established guidelines and standards [5,26,61]. Generally, synthetic excipients offer numerous advantages over natural counterparts, such as consistent quality, reproducibility, and cost-effectiveness [[94], [95], [96]]. The evaluation of the halal status of synthetic excipients is a complex, time-consuming, and costly process, which is further exacerbated by the lack of standardization among halal certification organizations. Despite these challenges, the development of halal-compliant synthetic excipients is increasingly becoming popular. This is driven by the desire to meet the needs of the Muslim population and potentially access new markets. Significant progress has been made in Certain porcine-derived products, such as heart valves, but insulin, cell-based therapies, peptides, and enzymes have proven difficult to replace [33,35,70].

The trend in drug development has shifted towards synthetic sources, departing from traditional animal and plant materials. This transition has yielded safer and more dependable products for patients. Specifically, synthetic insulin and heparin have supplanted their animal-derived counterparts due to concerns related to immune reactions and the associated long-term complications [97]. Patients have the fundamental right to make informed decisions about their medications, showing the need for accessible comprehensive information about the origins and composition of the drugs consumed [9,16,27].

### 3.3 Biotechnology

Several investigations have explored the development of halal-compliant microbial sources to produce pharmaceuticals that meet the needs of the Muslim community. This effort includes investigating halal-compliant bacteria, yeast, and fungi to generate proteins, enzymes, and vaccines. The use of these microbial sources offers notable advantages, including the capacity for large-scale production, precisely defined properties, and cost-effectiveness [[97], [98], [99]]. Moreover, cell-based systems, such as induced pluripotent stem cells (iPSCs) and mesenchymal stem cells (MSCs), have shown considerable potential in diverse fields, namely tissue engineering, regenerative medicine, and drug development [86,88].

Collagen is a widely used ingredient across various industries. However, its sourcing from non-halal origins has raised concerns within the Muslim community. To address this issue, attention has been shifted to recombinant collagen-like proteins as sustainable alternatives suitable for halal and vegetarian industries [36,63].

Based on observations, not all plant-based collagen can effectively replace animal-based collagen due to their differing characteristics. Scientific research has shown that bacteria-based collagen can be produced rapidly and in significant quantities, potentially rendering it a more cost-effective option compared to animal-derived products. Due to the growing demand for halal-certified products, the development of recombinant collagen-like proteins has become a viable solution for the halal industry, consistent with principles of sustainability and ethical sourcing. Furthermore, the application of recombinant collagen-like proteins extends to benefit the vegetarian community, adhering to their avoidance of animal-based products. As ongoing research continues to investigate the safety and efficacy of recombinant collagen-like proteins, their potential in several industries, including pharmaceutical and cosmetics, is anticipated to expand further [36,63].

### 3.4 Analogue products formulation

Several countries, including Malaysia, the United Arab Emirates, the United Kingdom, and Australia, have implemented regulations to ensure the compliance of pharmaceutical products with halal requirements. In Malaysia, the government has established a halal Pharmaceutical Task Force responsible for developing halal pharmaceutical standards and guidelines and providing certification. Similarly, the Emirates Authority for Standardization and Metrology (ESMA) in the United Arab Emirates (UAE) has implemented regulations to ensure that all pharmaceutical products imported or manufactured in the country adhere to halal standards. In the United States, the Food and Drug Administration (FDA) permits the use of porcine-derived ingredients in medications but mandates declaration through labeling by manufacturers [15,71]. The American Medical Association House of Delegates has passed a resolution recommending efforts to improve cultural awareness of animal-derived medication ingredients and urging the FDA to provide information about the source of medication ingredients accessible [41,47].

In response to the escalating demand for halal and kosher products, some pharmaceutical companies have started developing alternative formulations using plant-based or synthetic ingredients instead of porcine-derived components. Additionally, certain companies are striving to pioneer novel production methods without the use of animal-derived ingredients.

Manufacturers can instill robust confidence in consumers regarding the halal integrity of the final products by embracing best practices and enforcing regulations that require pharmaceutical companies to transparently disclose the ingredients used in their products. Through the development of alternative formulations, companies can cater to a broader spectrum of consumers while advocating for more sustainable and ethical production methods.

### 3.5 Alternatif drug and excepient

Halal pharmaceutical alternatives have been explored to meet the increasing demand for halal products among the world's second-largest population group, Muslims [[100], [101], [102], [103]]. Essential oils have been established as a viable therapeutic source due to their use in conventional medicine [12,20]. Although halal food is readily available, the consumption of pharmaceutical products depends on the condition of the patient, alternatives are essential [7,38,45].

In the context of substituting animal gelatin in recipes, there are several plant-based alternatives available that offer similar binding and gelling properties [26,36,104]. These alternatives include agar-agar, carrageenan, pectin, xanthan gum, and cellulose gum [[105], [106], [107]].

1) Agar-agar is a vegetable gelling extract from red algae commonly used in jams, fruit jellies, and puddings. This extract has almost no taste or color and is identified by the E406 code in the ingredients lists.
2) Carrageenan is a polysaccharide extract from red algae that is often used as a thickener, gelling agent, emulsifier, and stabilizer in the food industry. Furthermore, it has the E407 code for classifying food additives and is a powerful gelling agent capable of jellifying more than a litre of water with just a teaspoon.
3) Pectin is a plant-based substance abundant in various fruits such as gooseberries, apples, quinces, citrus seeds, and zest. This substance is identified by the E440 code in the ingredients list and is commonly used as a thickener, stabilizer, gelling agent, and emulsifier.
4) Xanthan gum is a polysaccharide obtained from the action of a bacterium, Xanthomonas campestris, and is used as a food additive under the code E415. Furthermore, it is soluble in cold water and is valued for its thickening, stabilizing, foaming, and gelling properties.
5) Cellulose gum is a white, slightly yellowish, or greyish powder that is odorless and tasteless. This powder is used as a food additive under the code E466 and is commonly applied as a thickener, stabilizer, and emulsifier.

## 4 Steps for substituting from animal-derived to halal derived

The transition to halal pharmaceuticals can commence by gaining a thorough comprehension of halal guidelines, regulations, and standards. This process is crucial to establish a precise definition of what constitutes halal and haram within the pharmaceutical industry. Manufacturers and research teams should also recognize the essential role played by halal certification organizations and the procedures in obtaining the certificate for pharmaceutical products.

### 4.1 Halal first from legal framework

The concept of halal, representing permissibility under Islamic law, has gained significant recognition in the global market, particularly in the food and pharmaceutical sectors [[108], [109], [110]]. This increased awareness originates from consumer concerns, specifically among Muslims, regarding the presence of non-halal ingredients in products [26,57,111,112].

Some countries, such as Malaysia, have implemented regulations governing the halal status of medicines and pharmaceutical products to ensure compliance with halal requirements [[113], [114], [115]]. However, several nations still lack the required guidelines and regulations concerning the use of animal-derived ingredients in medications [34,35]. The Malaysian Standard (MS) 2424 currently serves as the primary reference, emphasizing the need for a more comprehensive manual that pharmaceutical companies can follow to ensure strict adherence to halal requirements [116].

The guidelines and regulations regarding the use of animal-derived ingredients in pharmaceuticals have been established by several countries to address compliance issues, including the United Kingdom and Australia [19,34]. In the United States, the American Medical Association House of Delegates passed a resolution in 2018 advocating for initiatives to enhance cultural awareness regarding animal-derived medication ingredients. This resolution urges the FDA to enhance accessibility to information regarding the origin of medication ingredients [34].

Regulatory bodies play a significant role in establishing precise guidelines and regulations to guarantee the suitability of medications for consumption by Muslim consumers due to the increasing significance of the halal economy and the substantial global Muslim population [108,109]. Furthermore, the introduction of regulations mandating pharmaceutical companies to transparently declare the ingredients used in their products can instill confidence in consumers regarding the halal integrity of the final products [63,[117], [118], [119]].

The Standards and Metrology Institute for the Islamic Countries (SMIIC) has recently released three new Halal Standards. These include Organization of Islamic Cooperation (OIC)/SMIIC 57:2022 for halal inspection bodies, OIC/SMIIC 50–1:2022 for halal pharmaceuticals, and OIC/SMIIC 23:2022 for halal animal feed. Furthermore, these standards establish requisites for inspecting, manufacturing, transporting, and preparing halal products to ensure compliance with Islamic principles and global consumer expectations. SMIIC remains steadfast in its commitment to the global advancement of high-quality halal standards [19]. The existence of diverse halal standards presents a challenge to global recognition, hindering trade and creating complexities for manufacturers striving to comply. This problem can be overcome through the harmonization of monitoring systems and the establishment of a unified global halal standard. Collaborative efforts among authorities worldwide are also indispensable in crafting a uniform regulatory framework and standardized certification procedures in the global market. Although the Menteri Agama Brunei Darussalam, Indonesia, Malaysia, Singapura (MABIMS) initiative has shown positive outcomes in monitoring halal certification implementation, particularly in the context of pharmaceutical products, warranty enhancements are still required [43]. Moreover, pursuing a universally recognized global halal standard can stimulate trade and instill confidence in both producers and consumers, facilitating the expansion of the halal industry [120].

The main objective of regulations is to ensure that consumers have access to accurate and truthful information regarding products acquired. These regulations ensure that products are genuinely halal and safe for consumption. Through collaborative efforts to establish clear guidelines and regulations, the needs and concerns of Muslim consumers can be effectively achieved, fostering trust within the halal market.

### 4.2 Exploring novel proteinaceous ingredients

The pharmaceutical industry is experiencing an increasing demand for halal pharmaceutical products that adhere to Islamic dietary laws [29,118,121]. To meet this demand, there is a need to explore novel halal proteinaceous ingredients that can replace animal-derived ingredients in medication [[122], [123], [124], [125]]. These ingredients can be obtained from soy, peas, and beans or microbial sources such as bacteria and fungi [104,[126], [127], [128], [129], [130]].

Pea protein has shown promising potential as a plant-based ingredients with functional properties similar to animal-derived gelatin [[131], [132], [133], [134]]. Furthermore, it can be used in various pharmaceutical applications, including capsule and tablet coating production. Soy protein has also been researched as a potential alternative to gelatin for soft gel capsule manufacturing [135,136].

Microbial sources are currently being investigated for halal pharmaceutical ingredients [85,[137], [138], [139]]. For example, bacterial fermentation can produce recombinant proteins capable of replacing animal-derived ingredients in medications. Fungal sources, such as mycelium, have the potential to produce protein-based materials suitable for pharmaceutical use [53,138,[140], [141], [142]].

In addition to exploring novel proteinaceous ingredients, there is a need to establish guidelines and regulations for using these ingredients in pharmaceutical products. In Malaysia, regulatory bodies, such as the FDA and the Halal Pharmaceutical Task Force, play an important role in establishing these guidelines and ensuring the compliance of pharmaceutical products with halal requirements. By promoting the use of halal ingredients and establishing clear guidelines, the pharmaceutical industry can meet the growing demand for halal pharmaceutical products while promoting more sustainable and ethical production methods**.**

### 4.3 Functionality of protein sources

Protein is an essential nutrient that plays a crucial role in the growth and maintenance of tissues in the human body [143]. Furthermore, it comprises chains of amino acids found in various food sources, including animal and plant-based products [104,[126], [127], [128], [129], [130]]. In halal pharmaceuticals, the selection of appropriate protein sources is important to ensure both halal compliance and functionality.

Historically, animal-based proteins such as collagen, gelatin, and albumin have found extensive use in pharmaceuticals due to their functional attributes. For example, collagen and gelatin are commonly used in the production of capsules, coatings, and gels due to their capacity to form stable matrices. Albumin also serves as a stabilizer and a source of amino acids in intravenous solutions. However, these ingredients are derived from non-halal sources, such as porcine and bovine, presenting a challenge for halal pharmaceuticals.

Plant-based protein sources, including soy, pea, and rice, have gained prominence in recent years due to their functional properties and halal status [[131], [132], [133], [134]]. Soy protein has emulsifying properties and can be used to create suspensions, emulsions, and foams [104,144,145]. Pea protein possesses excellent water-holding properties, making it suitable as a thickener and gelling agent [[146], [147], [148], [149]]. Furthermore, rice protein frequently serves as a stabilizer and a source of amino acids in pharmaceutical applications [126,150,151].

Beyond their functional attributes, plant-based protein sources offer several advantages compared to animal-based counterparts. Plant-based protein is more environmentally sustainable, requiring fewer resources for production, and is often cost-effective. Moreover, these protein sources are devoid of many allergens commonly associated with animal-based proteins, such as lactose and gluten [133,134].

### 4.4 Detection methods

The contamination of food and pharmaceutical products with porcine ingredients has been a growing concern globally [26,152]. In 2008, a blood-thinning drug called heparin was recalled in the United States after being contaminated with oversulfated chondroitin sulfate (OSCS), a substance derived from pig cartilage. This incident led to increased efforts to improve quality control and traceability of pharmaceutical ingredients [153,154].

In 2013, a scandal unfolded in Europe when horsemeat was sold as beef, leading to a comprehensive investigation into the food industry [155,156]. A major food company in China was found to have sold expired and contaminated meat products, including chicken and beef, that contained traces of pork [154,155,157].

In 2018, a blood pressure medication was found to be contaminated with a carcinogen called *N*-nitroso dimethylamine (NDMA). This contamination was traced back to the use of porcine waste products in the manufacturing process. These incidents have shown the importance of effective detection methods and quality control measures to prevent contamination of products with porcine ingredients [158,159]. The analytical steps for testing the halalness of cosmetics include the preparation and extraction of lard components using various methods, as well as the acquisition and analysis of Fourier-transform infrared (FTIR) spectra [160,161].

Aluwi (2017) further suggested that regulations such as Law No. 33/2014 on Halal Products Assurance should require labeling halal products and those containing non-halal ingredients such as pig fragments and blood [162,163]. This method is similar to labeling the percentage of alcohol in syrups that contain alcohol. Moreover, recent updates in detection methods for porcine materials in drugs have focused on improving accuracy, sensitivity, and reliability. DNA-based methods are highly sensitive and specific, enabling the detection of trace amounts of porcine DNA in complex matrices such as pharmaceutical products [161,[164], [165], [166], [167]]. However, these methods require specialized equipment and expertise, making the process relatively time-consuming and expensive compared to other methods, such as enzyme-linked immunosorbent assay (ELISA) and FTIR [168,169]. The methods remain valuable tools for ensuring compliance with halal standards for Muslim consumers.

The liquid chromatography-tandem mass spectrometry (LC-MS/MS) method is capable of detecting pork and beef DNA at low concentrations of 0.005–0.1 % [170]. This method has great potential for quality control in the pharmaceutical industry. Another research developed a polymerase chain reaction (PCR)-based assay that detected porcine DNA in heparin sodium, a widely used anticoagulant drug, at a lower concentration of 0.01 % [171,172]. The LC-MS-based metabolomic and lipidomic methodology has shown excellent sensitivity in describing the fingerprint of metabolites as well as lipids in pork and beef [173].

Immunoassay-based methods, such as the lateral flow immunoassay (LFIA), have also been used to detect porcine-specific proteins in pharmaceutical products. These methods are useful for rapidly detecting porcine contamination in large batches of products [[174], [175], [176], [177]].

### 4.5 Halal directory

The halal directory can provide significant value for Muslims in search of alternatives, spanning medications, food products, and consumer goods. In Indonesia, there is a pressing need for the establishment of precise guidelines and standards governing the intake and production of halal medications. This ensures that patients can make well-informed decisions regarding their health while adhering to religious beliefs. The availability of a comprehensive catalog detailing halal and non-halal medications should also be easily accessible to both healthcare providers and recipients. Furthermore, pharmaceutical companies must be held accountable to uphold these standards, fostering trust and confidence among patients. The halal directory has shown promising potential to promote halal businesses and incentivize more companies to pursue halal certification, thereby expanding the accessibility of regularized products and services [178]. When coupled with initiatives aimed at maintaining transparency in labeling and distribution, as well as facilitating open communication between healthcare providers and pharmaceutical companies, the halal directory can create an ideal environment within the halal community.

### 4.6 Practitioner

The recent increase in the use of alternative medication by patients for self-care shows the importance for healthcare providers to inquire about their usage during reconciliation [[179], [180], [181], [182]]. Commonly prescribed drugs may also contain animal products, which can be neglected when reading ingredients [34,35]. Pharmacists may not always be well-versed in halal medication alternatives, showing the need for readily available resources to ensure the religious beliefs of patients are respected and accommodated [62,183,184]. In some cases, certain pharmaceutical drugs receive conditional certification based on the principle of necessity. However, a previous report shows that pharmacists remain unaware of halal medications or alternative options for patients with dietary restrictions [7,185]. This shows the importance of increasing awareness among medical professionals and patients as well as the provision of accessible resources for locating and using halal pharmaceuticals.

**Fig. 6:**
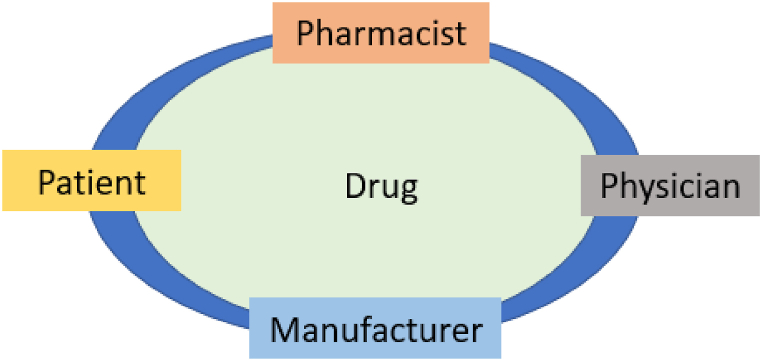
Role of Practitioners in drug dispensing.

In the halal pharmaceutical industry, informed consent takes on a legal dimension, as patients are not always aware of the presence of non-halal ingredients in their prescribed medications. Consequently, pharmacists should possess a thorough understanding of medication ingredients and address these concerns with patients based on their autonomy. Although a list of common medications for patients with dietary restrictions is useful for healthcare providers, manufacturers are not always capable of ascertaining the source of excipients [62]. The more informed pharmacists are regarding these issues, the better their understanding of pertinent questions on the consideration of religious beliefs and dietary restrictions of patients. This phenomenon can improve medication adherence and foster trust between healthcare professionals and their patients. Generally, there is a need to increase awareness among healthcare professionals and care recipients to ensure that appropriate resources are used to meet the needs of this underserved population, as presented in Fig. 6 [62].

This research showed that assessing the appropriateness of common drugs for individuals with specific dietary preferences can be challenging. Furthermore, it emphasized the importance of eliminating animal-derived components from medications wherever feasible. Some manufacturers have already started producing lactose without the use of rennet and producing magnesium stearate without animal ingredients [186]. Additionally, vegetarian capsules are useful as a substitute for gelatin. Halal Center at Airlangga University in Indonesia is actively working to facilitate the development of halal pharmaceutical products by establishing pharmaceutical research programs. These programs have the potential to yield positive results after receiving support from stakeholders who endorse the concept of substituting non-halal products with halal alternatives.

During the application of the Maqasid al-Shariah principle in pharmaceuticals, several specific conditions must be taken into consideration. Firstly, the situation should entail a life-threatening condition, where pharmaceuticals are essential for preserving life or preventing severe harm. Secondly, there should be no readily available halal alternatives effective for treating the condition or providing the necessary cure. Thirdly, a qualified Muslim doctor must recommend the use of pharmaceuticals, considering their expertise and understanding of the condition of the patient. Finally, there should be a reasonable certainty that the selected pharmaceutical products will act as a source of cure or significantly alleviate the life-threatening condition. These conditions establish a framework for determining the permissibility of non-halal pharmaceuticals in critical situations, ensuring the preservation of life while adhering to Islamic ethical considerations. Furthermore, it is essential to seek guidance from knowledgeable scholars or authorities to address individual cases due to variations in interpretations depending on circumstances and cultural context [9,21,67].

### 4.7 Consumer

Halal products have experienced increasing popularity among consumers who prioritize safety, hygiene, and high-quality standards in their consumption options [187,188]. This trend can be attributed to the Islamic dietary restrictions prohibiting pork or any pork-deitary [7,35,65]. Considering the rapid increase in the Muslim population globally, reaching approximately 1.6 billion people [189,190], there is a need for the medical community to gain a comprehensive understanding of the specific medical requirements of this diverse ethnic group, rived ingredients, and making halal products a preferred option for Muslims Healthcare providers must also consider patient-specific needs to effectively cater to the healthcare requirements of Muslim patients [[191], [192], [193]]. Consequently, it is crucial to recognize the significance of halal products and their significant role in safeguarding the well-being of the Muslim community. To build consumer awareness about halal pharmaceutical products, several actions are required:

1) Increase education about halal and non-halal pharmaceutical products [63].
2) Make halal certification clear and easily accessible to consumers [46,194].
3) Encourage the pharmaceutical industry to follow halal standards set by the authorities [7,81].
4) Promote halal pharmaceutical products available in the market [39,194].
5) Encourage consumers to check or verify pharmaceutical products before purchasing them [195].
6) Provide complaints or mechanisms to report suspected non-halal pharmaceutical products.

## 5 Conclusions and perspective

This research showed the need for collaborative efforts among regulatory authorities, healthcare professionals, and pharmaceutical manufacturers to address the availability of halal alternatives for pharmaceutical ingredients. Furthermore, the importance of raising awareness regarding halal and non-halal considerations was emphasized. Through joint endeavors, these stakeholders can establish guidelines and foster the development of halal pharmaceuticals. The provision of adequate education for both professionals and consumers would also encourage the adoption of halal alternatives sourced from permissible origins, effectively addressing concerns and meeting the requirements of Muslim consumers [7,120,196].

The government played a significant role in establishing robust governance for existing halal regulations. The implementation of a regulatory enforcement system can effectively promote compliance among all stakeholders, with specific attention to the pharmaceutical industry. The government can expedite the advancement of research into alternative materials. Although certain countries had already laid down comprehensive certification guidelines, there remained a necessity to bolster and broaden these regulations consistent with increasing reliance on pharmaceutical products [14,93].

The implementation of a halal management system in the pharmaceutical industry would facilitate adequate compliance with halal guidelines and regulations throughout the entire product development, manufacturing, and distribution process [197,198].

Collaboration among research teams, producers, Islamic scholars, and the Muslim community is significantly important in the development, testing, evaluation, and commercialization of various halal research initiatives [[199], [200], [201]]. Several research studies have yielded promising results, particularly in terms of vegetable-based excipients that are in line with halal requirements. This field of research has gained increasing attention as a burgeoning within the industry, addressing the growing demand for products that adhere to halal standards. Another crucial step towards transforming into halal pharmaceutical entails the creation of alternatives for excipients derived from pork and other non-compliant sources. These alternatives include plant-based, microbial-based, or synthetic excipients that fully conform to halal guidelines and regulations. The development of halal-compliant cell-based system can be a complex, time-consuming, and costly endeavor, fraught with ethical and safety considerations. Although the global market for cell-based therapeutics is poised for growth, the development is still ongoing. These efforts ensure that the products developed are consistent with the needs and expectations of the Muslim community.

Healthcare providers must remain well-informed about available alternatives and effectively communicate with patients to gain insight into their preferences and beliefs. This research also showed insight into the lack of knowledge and awareness among pharmacists regarding halal medications and dietary restrictions. Furthermore, there was a pressing need for readily accessible resources that could assist pharmacists in determining the suitability of medications for patients with religious or dietary constraints. A list of common medication alternatives permissible for patients with restrictions was also provided [62,191,193].

The shift towards halal pharmaceuticals was driven by increasing consumer demand for halal-compliant products and growing awareness among healthcare professionals as well as the general public [7,45].

Additional challenges related to pharmaceutical products include concerns regarding the use of products derived from Genetically Modified Organisms (GMOs), which intervene in the natural growth processes of living organisms. The intervention increased safety and compliance issues with halal guidelines, as some research reported unsafe and non-halal GMOs in several pharmaceutical products. To ensure the safety and compliance of these products with halal guidelines, there is a need to strengthen regulations and enforcement measures. Moreover, bolstering the research sector is crucial in providing alternative solutions to replace animal-derived products.

In conclusion, this research showed that the transition towards halal pharmaceuticals required collaboration among diverse stakeholders, including the government, research team, producers, Islamic scholars, and the Muslim community. The government played a significant role in enforcing compliance with halal regulations in the pharmaceutical industry. The development of halal alternatives for ingredients sourced from pork, such as plant-based or synthetic options, showed significant importance. Furthermore, healthcare providers should be knowledgeable about halal alternatives and maintain effective communication with patients to understand their preferences. Strengthening regulations and research related to GMOs was identified as essential to ensure safety and adherence to halal guidelines. Increasing awareness and meeting consumer demand was also important in the shift towards halal pharmaceuticals. This research mainly focused on excipients and might not comprehensively cover all aspects of halal alternatives in pharmaceuticals. Consequently, further investigation is recommended, exploring critical considerations, such as active ingredients or manufacturing processes.

## Funding

This research was funded by the Rector of 10.13039/501100015690Universitas Padjadjaran for Hibah Penulisan Review Artikel 2023 (No: 1549/UN6.3.1/PT.00/2023).

## Data availability statement

Data included in the article/supp. material/referenced in the article.

## Additional information

No additional information is available for this paper.

## CRediT authorship contribution statement

**Yedi Herdiana:** Writing – original draft, Visualization, Project administration, Formal analysis, Conceptualization. **Ferry Ferdiansyah Sofian:** Writing – review & editing, Methodology, Investigation, Data curation. **Shaharum Shamsuddin:** Writing – review & editing, Supervision. **Taofik Rusdiana:** Writing – original draft, Validation, Supervision, Software.

## Declaration of competing interest

The authors declare that they have no known competing financial interests or personal relationships that could have appeared to influence the work reported in this paper.

## References

[1] Irfany M.I., Rusydiana A.S. (2022). Challenges in developing integrated halal industry in Indonesia. Halal Tour. Pilgr..

[2] Bachtiar F.R. (2022). Halal certification of Indonesian cosmetics products : new protectionism and the rise of islamic populism in Indonesia. Nation State J. Int. Stud..

[3] Briliana V., Mursito N. (2017). Asia Paci fi c Management Review Exploring antecedents and consequences of Indonesian Muslim youths ’ attitude towards halal cosmetic products : a case study in Jakarta, Asia Pacific Manag. Rev.

[4] Shamsuddin A., Yusof F.M. (2020). Exploring the issues and challenges in Malaysian cosmetic halal: a theoretical framework. Nusant. Halal J..

[5] Sugibayashi K., Yusuf E., Todo H., Dahlizar S., Sakdiset P., Arce F.J., See G.L. (2019). Halal cosmetics: a review on ingredients, production, and testing methods. Cosmetics.

[6] Jaswir I., Sari D.P., bin Haji Che Daud M.R., Sukmana R. (2023). Motives for participation in halal food standard implementation: an empirical study in Malaysian halal food industry. Int. J. Islam. Middle E Finance Manag..

[7] Herdiana Y., Rusdiana T. (2022). Indonesian halal pharmaceutical: challenges and market opportunities. Indones. J. Pharm..

[8] Yani M.T., Suryaningsih S.A. (2021). Muslim consumer behavior and halal product consumption. J. Islam. Econ..

[9] Aziz N.A., Ibrahim I., Raof N.A. (2014). The need for legal intervention within the halal pharmaceutical industry. Procedia - Soc. Behav. Sci..

[10] Sahilah A.M., Fadly M.L., Norrakiah A.S., Aminah A., Wan Aida W., Ma’aruf A.G., Khan M.A. (2012). Halal market surveillance of soft and hard gel capsules in pharmaceutical products using PCR and southern-hybridization on the biochip analysis. Int. Food Res. J..

[11] RinaBintiRajaIkram R., Khanapi bin Abdul Ghani M., Samad Hasan Basari A. (2013). Novel computerized halal pharmaceuticals supply chain framework for warehouse and procurement. Int. J. Comput. Appl..

[12] Luxminarayan L., Neha S., Amit V., Khinchi M.P. (2017). Pork DNA contamination in pharmaceutical products: a review. Asian J. Pharmaceut. Res. Dev..

[13] Muhsin S.M. (2022). Medical confidentiality ethics: the genesis of an islamic juristic perspective. J. Relig. Health.

[14] Septiani D., Ridlwan A.A. (2022). The effects of halal certification and halal awareness on purchase intention of halal food products in Indonesia. Indones. J. Halal Res..

[15] Al-hakim A. (2021). General considerations for diversifying heparin drug products by improving the current heparin manufacturing process and reintroducing bovine sourced heparin to the US market. Clin. Appl. Thromb. Hemost..

[16] W. Paper, Position Paper on Indonesia Halal Policy for Medical Technologies an Industry Perspective,(n.d.)..

[17] Tayob S. (2021). Sustainability and Halal : Procedure , Profit and Ethical Practice.

[18] Rubio N.R., Xiang N., Kaplan D.L. (2020). Plant-based and cell-based approaches to meat production. Nat. Commun..

[19] Organization of Islamic Cooperation (2023). New Halal Standards.

[20] Abdullah N., Hazahari N.Y., Amid A. (2022). Essential oils as alternative halal therapeutic source for lung cancer: a mini review. Halalpshere.

[21] Kifli S.N., Kwen Fee L., Carnegie P.J., Hassan N.H. (2023). Re)Presenting Brunei Darussalam A Sociol. Everyday.

[22] Muqiita F., Darmawan G., Faturrohman M.S. (2023). The impact of awareness , certification , and quality of halal food on consumer purchase intentions at asia kintan buffet restaurant dampak kesadaran , sertifikasi , dan kualitas makanan halal terhadap niat pembelian konsumen restoran asia kintan buffet. J. Ekon. Syariah Teor. Dan Terap..

[23] Su I. (2020). Importance of ethnopharmacological studies in drug discovery : role of medicinal plants. Phytochemistry Rev..

[24] Bezerra F.F., Oliveira S.N.M.C.G., Sales R.A., Piquet A.A., V Capill N., Vilanova E., Tovar A.M.F., Mour P.A.S. (2023). Approaches to assure similarity between pharmaceutical heparins from two different manufacturers. Pharmaceutics.

[25] Shute J.K. (2023). Heparin , low molecular weight heparin , and non-anticoagulant derivatives for the treatment of inflammatory lung disease. Pharm. Times.

[26] Zin Z.M., Sarbon N.M., Zainol M.K., Jaafar S.N., Shukri M.M., Rahman A.H.A. (2021). Halal and non-halal gelatine as a potential animal by-products in food systems: prospects and challenges for Muslim community. Proc. First Int. Conf. Sci. Technol. Eng. Ind. Revolut. (ICSTEIR 2020).

[27] Statement G. (2015). Medicines/pharmaceuticals of animal origin. Queensl. Heal..

[28] Alam A.Y. (2017). The challenge of dealing with animal derived ingredients in medical/surgical products. J. Pakistan Med. Assoc..

[29] Saha Tushar, Rifat Tashnuva, Shimanto S. (2019). Prospects of halal pharmaceuticals asian journal of ethnopharmacology and medicinal foods prospects of halal pharmaceuticals. Asian J. Ethnopharmacol. Med. Foods..

[30] Pottel J., Armstrong D., Zou L., Fekete A., Huang X., Torosyan H., Bednarczyk D., Whitebread S., Bhhatarai B., Jin H., Ghaemi S.N., Slocum S., V Lukacs K., John J., Berg E.L., Giacomini K.M., Roth B.L., Shoichet B.K. (2021). The activities of drug inactive ingredients on biological targets. Science.

[31] Saito J., Agrawal A., Patravale V., Pandya A., Orubu S., Zhao M., Andrews G.P., Petit-turcotte C., Landry H., Croker A., Nakamura H. (2022). Perspectives of pharmaceutical excipients in pediatric patients in each country and region. Children.

[32] U.A.-Z. and Y.M. Haroon Latif (2016).

[33] Rodger D., Blackshaw B.P. (2019). Using animal-derived constituents in anaesthesia and surgery: the case for disclosing to patients. BMC Med. Ethics.

[34] Babos M.B., Perry J.D., Reed S.A., Bugariu S., Hill-Norby S., Allen M.J., Corwell T.K., Funck J.E., Kabir K.F., Sullivan K.A., Watson A.L., Kelli Wethington K. (2021). Animal-derived medications: cultural considerations and available alternatives. J. Osteopath. Med..

[35] Hassanein M., Anderson J.A. (2021). Refusal of animal-derived medical products in a paediatric setting: ethical issues. Paediatr. Child Health.

[36] Bapat V.A., Kishor P.B.K., Jalaja N., Jain S.M. (2023).

[37] Abdulhafiz F., Mohammed A., Farhan M., Reduan H., Abdul Z., Seong L., Wen K. (2022). Plant cell culture technologies : a promising alternatives to produce high-value secondary metabolites. Arab. J. Chem..

[38] Ningtyas P.F., Permana I., Rosa E.M., Jaswir I. (2022). Halal medicine selection process in sharia-certified hospital, Indonesia. J. Halal Res..

[39] Kasri R.A., Ahsan A., Widiatmoko D., Hati S.R.H. (2023). Intention to consume halal pharmaceutical products: evidence from Indonesia. J. Islam. Mark..

[40] Kadang L., Mutis A., Karyawati A.T. (2022). Halalan thayyiban chemistry: the topics of chemistry methods in food safety perspective. Nas. Kim. Dan.

[41] Randeree K. (2019). Challenges in halal food ecosystems: the case of the United Arab Emirates. Br. Food J..

[42] Almarzouqi F., Rennekampff H.-O., Almarzouki M., Lambertz A., Acharya M., Klink C., Popov A.-F., Pallua N. (2019). Porcine-derived biomaterials in tissue engineering and reconstructive surgery: considerations and alternatives in Muslim patients. J. Tissue Eng. Regen. Med..

[43] Abdallah A., Rahem M.A., Pasqualone A. (2021). The multiplicity of halal standards : a case study of application to slaughterhouses. J. Ethn. Foods..

[44] Abdullah F.A.A., Borilova G., Steinhauserova I. (2019). Halal criteria versus conventional slaughter technology. Animal.

[45] (2020). The Economist, Halal for health : scaling up halal pharmaceuticals. Intell. Unit..

[46] Sudarmiatin S., Khoirul Anam F., Wafaretta V. (2020). The intention of halal certification by micro business. KnE Soc. Sci..

[47] Sugita I. (2017). Halal in Singapore. Glob. Agric. Inf. Netw. (GAIN Report).

[48] Wijayant R., Kaukab M.E. (2019). Istihalah issue of halal food, medicine, and cosmetics. Med. Cosmet. J. Islam. Soc. Econ. Dev..

[49] Izhar Ariff Mohd Kashim M., Abdul Haris A.A., Mutalib S. Abd, Anuar N., Shahimi S. (2023). Scientific and Islamic perspectives in relation to the Halal status of cultured meat. Saudi J. Biol. Sci..

[50] Kamali M.H. (2021). Haram, permanence, and change: the principle of substance transformation (istihalah). Shariah Halal Ind.

[51] Katdare A., Chaubal M.V. (2006).

[52] Karahalil E. (2020). Principles of halal-compliant fermentations: microbial alternatives for the halal food industry. Trends Food Sci. Technol..

[53] Pham J.V., Yilma M.A., Feliz A., Majid M.T., Maffetone N., Walker J.R., Kim E., Cho H.J., Reynolds J.M., Song M.C., Park S.R., Yoon Y.J. (2019). A review of the microbial production of bioactive natural products and biologics. Front. Microbiol..

[54] Atanasov A.G., Zotchev S.B., Dirsch V.M., Orhan I.E., Banach M., Rollinger J.M., Barreca D., Weckwerth W., Bauer R., Bayer E.A., Majeed M., Bishayee A., Bochkov V., Bonn G.K., Braidy N., Bucar F., Cifuentes A., D'Onofrio G., Bodkin M., Diederich M., Dinkova-Kostova A.T., Efferth T., El Bairi K., Arkells N., Fan T.P., Fiebich B.L., Freissmuth M., Georgiev M.I., Gibbons S., Godfrey K.M., Gruber C.W., Heer J., Huber L.A., Ibanez E., Kijjoa A., Kiss A.K., Lu A., Macias F.A., Miller M.J.S., Mocan A., Müller R., Nicoletti F., Perry G., Pittalà V., Rastrelli L., Ristow M., Russo G.L., Silva A.S., Schuster D., Sheridan H., Skalicka-Woźniak K., Skaltsounis L., Sobarzo-Sánchez E., Bredt D.S., Stuppner H., Sureda A., Tzvetkov N.T., Vacca R.A., Aggarwal B.B., Battino M., Giampieri F., Wink M., Wolfender J.L., Xiao J., Yeung A.W.K., Lizard G., Popp M.A., Heinrich M., Berindan-Neagoe I., Stadler M., Daglia M., Verpoorte R., Supuran C.T. (2021). Natural products in drug discovery: advances and opportunities. Nat. Rev. Drug Discov..

[55] Frank M., annis Matthew G., Watkins (2019). Pharmacodynamic drug-drug interactions. Physiol. Behav..

[56] Belialov F. (2022). Drug classification for patients with comorbidities. J. Pharm. Policy Pract..

[57] Ab Halim M.A.B., Kashim M.I.A.B.M., Salleh M.M.M., Nordin N.B., Husni A.B.M. (2015). Halal Pharmaceuticals, Soc. Sci..

[58] Wahab S., Bolatito A.S. (2023).

[59] Ng P.C., Amy N., Ahmad S., Hanifah S.A. (2022). Recent advances in halal food authentication : challenges and strategies. J. Food Sci..

[60] Lee J.K. (2016). Degradation of properties and loss of nutrients in gelatin soft capsules the manufacturing process. Korea J. Packag. Sci. Technol..

[61] Zilhadia Z., Harahap Y., Jaswir I., Anwar E. (2022). Evaluation and characterization of hard-shell capsules formulated by using goatskin gelatin. Polymers.

[62] Butler L., Mai T., Santanello C. (2018). Assessing pharmacists' knowledge of halal medications to support the health beliefs of patients. Inov. Pharm..

[63] Duasa J., Fatimah S., Noor M., Mohd M.A., Thaker T., Rahman M.P. (2020). The recombinant collagen-like protein as animal-based collagen substitution: a qualitative study. Jcis.

[64] Brondz I. (2018). Why judaism and islam prohibit eating pork and consuming blood as a food. Voice Publ.

[65] Ismail K., Nordin M.F., Awang M.D., Baharum N.B. (2022). The consumers ’ unders tandings towards halal food products in. Al-Sirat..

[66] Herindar E. (2022). Maqashid sharia and the importance of consuming halal food products for Z Muslim generation. Halal Res.

[67] Sands D., Gangal M., Martin R.S. (2018). A comparison of liprotamase, a non-porcine pancreatic enzyme replacement therapy, to porcine extracted pancrelipase in a non- inferiority randomized clinical trial in patients with cystic fibrosis. Clinical-InvestigationInvestigation..

[68] Hryhorowicz S. (2020). Application of genetically engineered pigs in biomedical research 1. Genes.

[69] Perleberg C., Kind A., Schnieke A. (2018). Genetically engineered pigs as models for human disease. Dis. Model. Mech..

[70] Schook L.B., Collares T.V., Darfour-Oduro K.A., De A.K., Rund L.A., Schachtschneider K.M., Seixas F.K. (2015). Unraveling the swine genome: implications for human health. Annu. Rev. Anim. Biosci..

[71] Zettler S., Renner S., Kemter E., Hinrichs A., Klymiuk N., Backman M., Riedel E.O., Mueller C., Streckel E., Braun-Reichhart C., Martins A.S., Kurome M., Keßler B., Zakhartchenko V., Flenkenthaler F., Arnold G.J., Fröhlich T., Blum H., Blutke A., Wanke R., Wolf E. (2020). A decade of experience with genetically tailored pig models for diabetes and metabolic research. Anim. Reprod..

[72] Hirsch I.B., Juneja R., Beals J.M., Antalis C.J., Wright E.E. (2020). The evolution of insulin and how it informs therapy and treatment choices. Endocr. Rev..

[73] Wang H. (2021). A review of the effects of collagen treatment in clinical studies. Polymers.

[74] Maurer T., Stoffel M.H., Belyaev Y., Stiefel N.G., Vidondo B., Kuker S., Mogel H., Schafer B., Balmer J. (2018). Structural characterization of four different naturally occurring porcine collagen membranes suitable for medical applications. PLoS One.

[75] Stroebinger N., Rutherfurd S.M., Henare S.J., Hernandez J.F.P., Moughan P.J. (2021). Fatty acids from different fat sources and dietary calcium concentration differentially affect fecal soap formation in growing pigs. J. Nutr..

[76] Talevi A., Bellera C.L. (2020). Expert Opinion on Drug Discovery Challenges and opportunities with drug repurposing : finding strategies to find alternative uses of therapeutics. Expet Opin. Drug Discov..

[77] Maclean N., Khadra I., Mann J., Abbott A., Mead H., Markl D. (2023). Formulation-dependent stability mechanisms affecting dissolution performance of directly compressed griseofulvin tablets. Int. J. Pharm..

[78] Ahirwar K., Shukla R., Shukla D.R., Kuznetsov P.A., Ali A.P.A. (2023). Drug Formul. Des..

[79] Douglas C.M.W., Aith F., Boon W., Borba M.D.N., Doganova L., Grunebaum S., Hagendijk R., Lynd L., Mallard A., Mohamed F.A., Moors E. (2022). Social pharmaceutical innovation and alternative forms of research, development and deployment for drugs for rare diseases. Orphanet J. Rare Dis..

[80] Patel R., Barker J. (2020). Pharmaceutical excipients and drug metabolism : a mini-review. Int. J. Mol. Sci..

[81] Tadauchi T., Yamada D., Koide Y., Yamada M., Shimada Y., Yamazoe E. (2022). Improving the powder properties of an active pharmaceutical ingredient (ethenzamide) with a silica nanoparticle coating for direct compaction into tablets. Powders.

[82] Hole G., Hole A.S., Mcfalone-shaw I. (2021). International Journal of Pharmaceutics : X Digitalization in pharmaceutical industry : what to focus on under the digital implementation process. Int. J. Pharm. X..

[83] Bin Yeom S., Choi D.H. (2019). Scale-up strategy in quality by design approach for pharmaceutical blending process with discrete element method simulation. Pharmaceutics.

[84] Jang E.H., Park Y.S., Kim M.S., Choi D.H. (2020). Model-based scale-up methodologies for pharmaceutical granulation. Pharmaceutics.

[85] Kurniati K., Hafsan H. (2022). Halal critical point of microbial bioprocess based-dairy products. J. Islam Sci..

[86] Wikandari R., Baldermann S., Ningrum A. (2021). Application of cell culture technology and genetic engineering for production of future foods and crop improvement to strengthen food security. Bioengineered.

[87] Refaie A.F., Elbassiouny B.L., Kloc M., Sabek O.M., Khater S.M., Ismail A.M., Mohamed R.H., Ghoneim M.A. (2021). From mesenchymal stromal/stem cells to insulin-producing cells: immunological considerations. Front. Immunol..

[88] Silva I.B.B., Kimura C.H., Colantoni V.P., Sogayar M.C. (2022). Stem cells differentiation into insulin-producing cells (IPCs): recent advances and current challenges. Stem Cell Res. Ther..

[89] Srinivasan S., Durairaj B. (2021). Collagen isolation and characterization from Sardinella longiceps. J. Adv. Vet. Anim. Res..

[90] Rakhmanova A., Khan Z.A., Sharif R., Lü X. (2018). Meeting the requirements of halal gelatin: a mini review. MOJ Food Process. Technol..

[91] Milovanovic I., Hayes M. (2018). Marine gelatine from rest raw materials. Appl. Sci..

[92] Veeresham C. (2012). Natural products derived from plants as a source of drugs. \"J. Adv. Pharm. Technol. Research\"\" (JAPTR)\".

[93] Mabkhot H. (2023). Factors affecting the sustainability of halal product performance: Malaysian evidence. Sustain. Times.

[94] Narang A.S., V Mantri R., Raghavan K.S., Qiu Y., Chen Y., Zhang G.G.Z., Yu L., R.V.B.T.-D.S.O.D.F. (Second E. Mantri (2017). Dev. Solid Oral Dos. Forms.

[95] Singh H., Khurana L.K., Singh R., Vohora D., G.B.T P.M., Singh T.C.R. (2018). Pharm. Med. Transl. Clin. Res..

[96] Kar M., Chourasiya Y., Maheshwari R., Tekade R.K., R.K.B.T.-B.F. of D.D. Tekade (2019). Chapter 2 - Current Developments in Excipient Science: Implication of Quantitative Selection of Each Excipient in Product Development.

[97] Choi D.H., Lee D., Jo B.S., Park K.S., Lee K.E., Choi J.K., Park Y.J., Lee J.Y., Park Y.S. (2020). A synthetic cell-penetrating heparin-binding peptide derived from BMP4 with anti-inflammatory and chondrogenic functions for the treatment of arthritis. Int. J. Mol. Sci..

[98] Raveendran S., Parameswaran B., Ummalyma S.B., Abraham A., Mathew A.K., Madhavan A., Rebello S., Pandey A. (2018). Applications of microbial enzymes in food industry. Food Technol. Biotechnol..

[99] Wehrs M., Tanjore D., Eng T., Lievense J., Pray T.R., Mukhopadhyay A. (2019). Engineering robust production microbes for large-scale cultivation. Trends Microbiol..

[100] Adah Muhamad N.S., Sulaiman S., Adham K.A., Said M.F. (2019). Halal Tourism: literature synthesis and direction for future research. Pertanika J. Soc. Sci. Humanit..

[101] Atalan-Helicke N. (2015). The halal paradox: negotiating identity, religious values, and genetically engineered food in Turkey. Agric. Hum. Val..

[102] Mazlan A.I., Hamzah H.Z. (2015). Malaysian halal export market : case study on developing countries. Persidang. Kebangs. Ekon. Malaysia Ke-.

[103] Pacific A. (2010). Global halal industry : an overview. Glob. Islam. Financ. Rep. 2013.

[104] Kyriakopoulou K., Keppler J.K., van der Goot A.J. (2021). Functionality of ingredients and additives in plant-based meat analogues. Foods.

[105] Saha D., Bhattacharya S. (2010). Hydrocolloids as thickening and gelling agents in food: a critical review. J. Food Sci. Technol..

[106] Hafezi K. (2022). Hydrocolloids: structure, preparation method, and application in food and pharmaceutical industries. Res. Strat..

[107] Marimuthu M., Ilansuriyan P., Yap T.N., Shanmugam M. (2017). Interaction of semi-refined carrageenan (E407A) with nano quanta of some food hydrocolloids and their physiochemical, functional and rheological properties. J. Microbiol. Biotechnol. Food Sci..

[108] Bergeaud-Blackler F., Fischer J., Lever J. (2015). Halal matters: islam, politics and markets in global perspective, halal matters islam. Polit. Mark. Glob. Perspect..

[109] Nafis M.C. (2019). The concept of halal and tayyib and its implementation in Indonesia. J. Halal Prod. Res..

[110] Susilowati I., Edy Riyanto E., Kirana M., Mafruhah I., Radam A. (2018). The economic and sharia value of moslem's awareness for halal food in Indonesia. J. Ekon. Pembang. Kaji. Masal. Ekon. Dan Pembang..

[111] Billah A., Rahman A., Bin Hossain T. (2020). Factors influencing Muslim and non-Muslim consumers ’ consumption behavior : a case study on halal food. J. Foodserv. Bus. Res..

[112] Damit D.H.D.A., Harun A., Martin D. (2017). Key challenges and issues consumer face in consuming halal product. Int. J. Acad. Res. Bus. Soc. Sci..

[113] Abdul Aziz N., Abdullah S.M., Nasrun M., Roslan M.A., Awang @ Ali M.N. (2022). Halal labelling for the Malaysian pharmaceutical products: a legal review. Int. J. Acad. Res. Bus. Soc. Sci..

[114] Draman W.M.A.A.W., Chun-Phuoc J., Yajid M.S.A. (2019). Halal certification of medical device in Malaysia: a shariah analysis into the compliance process and legal requirements. Int. J. Med. Toxicol. Leg. Med..

[115] Muhammad M.A., Elistina A.B., Ahmad S. (2020). The challenges faced by halal certification authorities in managing the halal certification process in Malaysia. Food Res..

[116] Latiff J.A., Zakaria Z., Man S. (2021). The challenges in implementation of halal vaccine certification in Malaysia. J. Food Pharm. Sci..

[117] Mohd Nawi N., Mohd Nasir N.I. (2014). Consumers' attitude toward the food safety certificate (FSC) in Malaysia. J. Food Prod. Market..

[118] Nor N.F., Ahmad H., Ariffin A.S. (2022). Confidence level to purchase halal food products via ordering online application. Proc. 6th Int. Conf. Food, Agric. Nat. Resour. (IC-FANRES 2021).

[119] Wan Sulong W.M., Husain S., Ismail M.Z., Othman M.S., Mohd Zin Z., Mohd Ghazali R. (2020). Halal food facilities in Japan from the perspective of Malaysian Muslim tourists. Int. J. Acad. Res. Bus. Soc. Sci..

[120] Fadliyah H., Nurwahyuni A. (2022). Policy implementation of halal product assurance for pharmaceutical products in Indonesia. J. Indones. Heal. Policy Adm..

[121] Rusmita S.A., Ryandono M.N.H., Filianti D., Mohd Salleh M.C. (2020). Islamic economic students knowledge and attitude toward halal pharmacy product in east java, Indonesia. Al-Uqud J. Islam. Econ..

[122] Baune M.C., Terjung N., Tülbek M.Ç., Boukid F. (2022). Textured vegetable proteins (TVP): future foods standing on their merits as meat alternatives. Futur. Foods.

[123] Ismail I., Hwang Y.H., Joo S.T. (2020). Meat analog as future food: a review. J. Anim. Sci. Technol..

[124] Syed Fazal ur Rahim (2022). Muhammad Abdullah Bin Masood, Global view of animal feed in halal perspective. GSC Adv. Res. Rev..

[125] Singh A., Sit N. (2022). Meat analogues: types, methods of production and their effect on attributes of developed meat analogues. Food Bioprocess Technol..

[126] Kumar M., Tomar M., Punia S., Dhakane-Lad J., Dhumal S., Changan S., Senapathy M., Berwal M.K., Sampathrajan V., Sayed A.A.S., Chandran D., Pandiselvam R., Rais N., Mahato D.K., Udikeri S.S., Satankar V., Anitha T., Reetu Radha, Singh S., Amarowicz R., Kennedy J.F. (2022). Plant-based proteins and their multifaceted industrial applications. Lwt.

[127] Molfetta M., Morais E.G., Barreira L., Bruno G.L., Porcelli F., Dugat-Bony E., Bonnarme P., Minervini F. (2022). Protein sources alternative to meat: state of the art and involvement of fermentation. Foods.

[128] Yong S., Sim J., Srv A., Chiang J.H. (2021). Plant Proteins for Future Foods : A Roadmap, Foods.

[129] Cichońska P., Ziarno M. (2022). Legumes and legume-based beverages fermented with lactic acid bacteria as a potential carrier of probiotics and prebiotics. Microorganisms.

[130] Ahnan-Winarno A.D., Cordeiro L., Winarno F.G., Gibbons J., Xiao H. (2021). Tempeh: a semicentennial review on its health benefits, fermentation, safety, processing, sustainability, and affordability. Compr. Rev. Food Sci. Food Saf..

[131] Boukid F., Rosell C.M., Castellari M. (2021). Pea protein ingredients: a mainstream ingredient to (re)formulate innovative foods and beverages. Trends Food Sci. Technol..

[132] Vargel C. (2020). Food industry, Corros. Alum.

[133] Hertzler S.R., Lieblein-Boff J.C., Weiler M., Allgeier C. (2020). Plant proteins: assessing their nutritional quality and effects on health and physical function. Nutrients.

[134] Masiá C., Jensen P.E., Petersen I.L., Buldo P. (2022). Design of a functional pea protein matrix for fermented plant-based cheese. Foods.

[135] Wilson H.T., Amirkhani M., Taylor A.G. (2018). Evaluation of gelatin as a biostimulant seed treatment to improve plant performance. Front. Plant Sci..

[136] Wulandari D., Erwanto Y., Pranoto Y., Rusman, Yuliatmo R. (2019). Improvement of Bovine Split Hide Gelatin quality by addition of soy protein isolate using transglutaminase enzyme. Trop. Anim. Sci. J..

[137] Kim B.H., Heo J., Park J. (2021). Determination of the 3D atomic structures of nanoparticles. Small Sci.

[138] Yap C.K., Al-Mutairi K.A. (2023). Effective microorganisms as halal -based sources for biofertilizer production and some socio-economic insights. Foods.

[139] Faridah H.D., Sari S.K. (2019). Utilization of microorganism on the development of halal food based on biotechnology. J. Halal Prod. Res..

[140] Augustin M.A., Hartley C.J., Maloney G., Tyndall S. (2023). Innovation in precision fermentation for food ingredients. Crit. Rev. Food Sci. Nutr..

[141] Amara A.A., El-Baky N.A. (2023). Fungi as a source of edible proteins and animal feed. J. Fungi..

[142] Lübeck M., Lübeck P.S. (2022). Fungal cell factories for efficient and sustainable production of proteins and peptides. Microorganisms.

[143] Mæhre H.K., Dalheim L., Edvinsen G.K., Elvevoll E.O., Jensen I.J. (2018). Protein determination—method matters. Foods.

[144] Song R., Murphy M., Li C., Ting K., Soo C., Zheng Z. (2018). Current development of biodegradable polymeric materials for biomedical applications. Drug Des. Dev. Ther..

[145] Akbarian M., Khani A., Eghbalpour S., Uversky V.N. (2022). Bioactive peptides: synthesis, sources, applications, and proposed mechanisms of action. Int. J. Mol. Sci..

[146] Pam Ismail B., Senaratne-Lenagala L., Stube A., Brackenridge A. (2020). Protein demand: review of plant and animal proteins used in alternative protein product development and production. Anim. Front..

[147] Bühler J.M., Schlangen M., Möller A.C., Bruins M.E., van der Goot A.J. (2022). Starch in plant-based meat replacers: a new approach to using endogenous starch from cereals and legumes. Starch/Staerke.

[148] Patole S., Cheng L., Yang Z. (2022). Impact of incorporations of various polysaccharides on rheological and microstructural characteristics of heat-induced quinoa protein isolate gels. Food Biophys..

[149] Hou W., Long J., Hua Y., Chen Y., Kong X., Zhang C., Li X. (2022). Formation and characterization of solid fat mimetic based on pea protein isolate/polysaccharide emulsion gels. Front. Nutr..

[150] Miedzianka J., Walkowiak K., Zielińska-Dawidziak M., Zambrowicz A., Wolny S., Kita A. (2023). The functional and physicochemical properties of rice protein concentrate subjected to acetylation. Molecules.

[151] Małecki J., Muszyński S., Sołowiej B.G. (2021). Proteins in food systems—bionanomaterials, conventional and unconventional sources, functional properties, and development opportunities. Polymers.

[152] Haji M., Kerbache L., Al-Ansari T. (2022). Food quality, drug safety, and increasing public health measures in. Supply Chain Management, Processes.

[153] (2018). US FDA, FDA encourages reintroduction of bovine-sourced heparin. US Food Drug Adm.

[154] Devlin A.J., Mycroft-West C.J., Turnbull J.E., de Lima M.A., Guerrini M., Yates E.A., Skidmore M.A. (2022). Analysis of heparin samples by attenuated total reflectance fourier-transform infrared spectroscopy in the solid state. ACS Cent. Sci..

[155] Rubio Lozano M.S., Hernández Chávez J.F., Ruíz López F.A., Medina Medina R., Delgado Suárez E., Méndez Medina R.D., Ngapo T.M. (2020). Horse meat sold as beef and consequent clenbuterol residues in the unregulated Mexican marketplace. Food Control.

[156] Smith R. (2019).

[157] Crceva-Nikolovska R., Angeleska A., Nikolovski A., Stojković-Dimitrievska E., Poposka-Treneska V., Sekovska B. (2019). Detecting meat fraud in food supply chain. West. Balk. J. Agric. Econ. Rural Dev..

[158] FDA (2020). https://www.fda.gov/news-events/press-announcements/fda-alerts-patients-and-health-care-professionals-nitrosamine-impurity-findings-certain-metformin.

[159] Mansouri I., Botton J., Semenzato L., Haddy N., Zureik M. (2022). N-nitrosodimethylamine-Contaminated valsartan and risk of cancer: a nationwide study of 1.4 million valsartan users. J. Am. Heart Assoc..

[160] Aziz A.A., Nordin F.N.M., Zakaria Z., Abu Bakar N.K. (2022). A systematic literature review on the current detection tools for authentication analysis of cosmetic ingredients. J. Cosmet. Dermatol..

[161] Kim Y.S., Yu H.K., Lee B.Z., Hong K.W. (2018). Effect of DNA extraction methods on the detection of porcine ingredients in halal cosmetics using real-time PCR. Appl. Biol. Chem..

[162] Hasan M.R. (2022). The legal regulation of halal product guarantees in Indonesia. JURE Crit. Laws J..

[163] Purwanto H., Jati S., Rofiq A. (2021). Policy analysis of enforcement of halal product guarantee regulations through the regulatory impact analysis (RIA) approach. J. Digit. Mark. Halal Ind..

[164] Muflihah A. Hardianto, Kusumaningtyas P., Prabowo S., Hartati Y.W. (2023). DNA-based detection of pork content in food. Heliyon.

[165] Amin R., Karim R.M., Rahman M.Z. (2017). http://www.bjimb.org.

[166] Shahimi S., Mutalib S. Abd, Ismail N., Elias A., Hashim H., Kashim M.I.A.M. (2021). Species-specific identification of porcine blood plasma in heat-treated chicken meatballs. Saudi J. Biol. Sci..

[167] Yusop M.H.M., Bakar M.F.A. (2020). Review on halal forensic: a focus on dna-based methods for pork authentication. Food Res..

[168] Alves Melo I.M., Pereira Viana M.R., Pupin B., Bhattacharjee T.T., de Azevedo Canevari R. (2021). PCR-RFLP and FTIR-based detection of high-risk human papilloma virus for cervical cancer screening and prevention. Biochem. Biophys. Reports..

[169] Zakaria N.D., Hamzah H.H., Salih I.L., Balakrishnan V., Abdul Razak K. (2023). A review of detection methods for vancomycin-resistant enterococci (VRE) genes: from conventional approaches to potentially electrochemical DNA biosensors. Biosensors.

[170] He L., Ma J., Li Q., Wang L., Fan S., Zhang Y. (2022). Determination of ingredients in livestock and poultry meat based on liquid chromatography-tandem mass spectrometry. J. Funct.Foods.

[171] Pecorini S., Camurri G., Torrini L., Ferraresi R. (2020). Highly sensitive real-time PCR method to identify species origin in heparinoids. Anal. Bioanal. Chem..

[172] Devlin A., Mycroft-West C., Procter P., Cooper L., Guimond S., Lima M., Yates E., Skidmore M. (2019). Tools for the quality control of pharmaceutical heparin. Méd..

[173] Harlina P.W., Maritha V., Musfiroh I., Huda S., Sukri N., Muchtaridi M. (2022). Possibilities of liquid chromatography mass spectrometry (LC-MS)-Based metabolomics and lipidomics in the authentication of meat products: a mini review. Food Sci. Anim. Resour..

[174] Andryukov B.G. (2020). Six decades of lateral flow immunoassay: from determining metabolic markers to diagnosing covid-19. AIMS Microbiol.

[175] Di Nardo F., Chiarello M., Cavalera S., Baggiani C., Anfossi L. (2021). Ten years of lateral flow immunoassay technique applications: trends, challenges, and future perspectives. Sensors.

[176] Zvereva E.A., Popravko D.S., Hendrickson O.D., Vostrikova N.L., Chernukha I.M., Dzantiev B.B., Zherdev A.V. (2020). Lateral flow immunoassay to detect the addition of beef, pork, lamb, and horse muscles in raw meat mixtures and finished meat products. Foods.

[177] Mirica A.C., Stan D., Chelcea I.C., Mihailescu C.M., Ofiteru A., Bocancia-Mateescu L.A. (2022). Latest trends in lateral flow immunoassay (LFIA) detection labels and conjugation process. Front. Bioeng. Biotechnol..

[178] Hasan K.N.S., Pasyah T. (2022). Challenges of Indonesian halal industry in the digital economic era. Sriwij. Law Rev..

[179] Butler J., Petrie M.C., Bains M., Bawtinheimer T., Code J., Levitch T. (2023). Challenges and opportunities for increasing patient involvement in heart failure self - care programs and self - care in the post–hospital discharge period. Res. Involv. Engagem..

[180] Rao M.T., Yamini M., Phanindra C.V.S., Rao Y.S. (2021). Alternative medicine: new ways to treat diseases and therapies. Indian J. Pharmaceut. Sci..

[181] Chautrakarn S., Khumros W., Phutrakool P. (2021). Self-medication with over-the-counter medicines among the working age population in metropolitan areas of Thailand. Front. Pharmacol..

[182] Plachkinova M., Kettering V., Chatterjee S. (2019). Increasing exposure to complementary and alternative medicine treatment options through the design of a social media tool. Heal. Syst..

[183] Syahrir A., Rahem A., Prayoga A. (2019). Pharmacist behavior of halal labelization on pharmaceutical product. J. Halal Prod. Res..

[184] Alserhan B.A., Bayirli M., Zakzouk F. (2020). Awareness towards Halal pharmaceuticals: an analysis of pharmacists' views. Int. J. Islam. Mark. Brand..

[185] Butler L., Mai T., Santanello C. (2018). Assessing pharmacists' knowledge of halal medications to support the health beliefs of patients. Innov Pharm.

[186] Tatham K.C., Patel K.P. (2014). Suitability of common drugs for patients who avoid animal products. BMJ Br. Med. J. (Clin. Res. Ed.).

[187] Purwanto A., Haque M.G., Sunarsih D., Asbari M. (2021). The role of brand image, food safety, awareness, certification on halal food purchase intention: an empirical study on Indonesian consumers. J. Ind. Eng. Manag. Res..

[188] Asnawi N., Sukoco B.M., Fanani M.A. (2018). Halal products consumption in international chain restaurants among global Muslim consumers. Int. J. Emerg. Mark..

[189] Grinin L.E. (2019). Islamism and globalization. J. Glob. Stud..

[190] Pew Research Center (2011). The future of the global Muslim population. Projections for 2010-2030, Popul. Sp. Place..

[191] Attum B., Hafiz S., Malik A., Shamoon Z. (2023). StatPearls, Treasure Island.

[192] Klitzman R., Di Sapia Natarelli G., Garbuzova E., Sinnappan S., Al-Hashimi J. (2023). Muslim patients in the U.S. confronting challenges regarding end-of-life and palliative care: the experiences and roles of hospital chaplains. BMC Palliat. Care.

[193] King J.K., Kieu A., El-Deyarbi M., Aljneibi N., Al-Shamsi S., Hashim M.J., Östlundh L., King K.E., King R.H., Ab Khan M., Govender R.D. (2023). Towards a better understanding between non-Muslim primary care clinicians and Muslim patients: a literature review intended to reduce health care inequities in Muslim patients. Heal. Policy OPEN..

[194] Wirdyaningsih I. Karimah, Syahida A.Q., Nabilah A.M. (2020). The optimization of halal certification in Indonesia: finding right balance between consumer and businessmen interest. Adv. Soc. Sci..

[195] Handono B.D., Sumaryono W., Saragi S. (2021). Analysis of customer behavior in purchasing decisions of medical services products and their effect on customer satisfaction in pharmacy. Int. J. Appl. Pharm..

[196] Ab Latiff J. (2020). Halal certification procedure in Malaysia and Indonesia. Petita J. Kaji. Ilmu Huk. Dan Syariah..

[197] Asmuni A., Jamil M., Rafianti F. (2020). Dynamics of application of halal certification on medicine products in Indonesia. Budapest Int. Res. Critics Inst. Humanit. Soc. Sci..

[198] Luthviati R.D., Jenvitchuwong S. (2021). Implementation of halal product assurance in the pharmaceutical sector in Indonesia. J. Hum. Rights, Cult. Leg. Syst..

[199] bin Salman A.M., Rohman N., Halim A., Susilayati M. (2019). Halal as a distinct competitive edge for islamic higher education in the millennial generation. Int. J. Halal Res..

[200] Nisa E.F. (2023). Transnational halal networks: INHART and the Islamic cultural economy in Malaysia and beyond. Global Network.

[201] Handayani D.I., Masudin I., Haris A., Restuputri D.P. (2022). Ensuring the halal integrity of the food supply chain through halal suppliers: a bibliometric review. J. Islam. Mark..

